# Integrative therapies for chronic insomnia: A randomized controlled trial of a traditional Thai herbal remedy and *Cannabis sativa* oil

**DOI:** 10.1016/j.sleepx.2026.100180

**Published:** 2026-02-17

**Authors:** Naruwat Pakdee, Nitcha Sribunrieng, Ronnachai Poowanna

**Affiliations:** a Department of Thai Traditional Medicine, Faculty of Natural Resources, Rajamangala University of Technology, Isan Sakon Nakhon Campus, Thailand

We sincerely thank the author for their thoughtful and rigorous critique and for highlighting important methodological and statistical issues in insomnia clinical trial research. We fully agree that methodological rigor is essential to ensure that clinical evidence meaningfully serves patients seeking effective and reliable treatments. We appreciate the opportunity to clarify the scope, design, and analytical framework of our study.

## 1 Choice of outcome measure (PSQI)

The Pittsburgh Sleep Quality Index (PSQI) was selected as the primary outcome measure because the primary objective of our study was to assess subjective sleep quality and patient-perceived improvement following integrative interventions. We acknowledge that the PSQI was originally developed as a general measure of sleep disturbance rather than an insomnia-specific diagnostic instrument. Our study was therefore not intended to serve as a diagnostic or mechanistic investigation of insomnia phenotypes, but rather as a pragmatic clinical evaluation of patient-reported sleep outcomes in individuals meeting DSM-5 criteria for chronic insomnia. The PSQI was used as a clinically relevant, validated, and widely applied patient-reported outcome measure to capture global sleep quality in real-world clinical settings.

## 2 Screening for comorbid sleep disorders

We acknowledge the importance of systematic screening for comorbid sleep disorders, including obstructive sleep apnea (OSA), in insomnia research. In the present study, formal polysomnographic screening or structured diagnostic assessment for OSA was not performed. Participant eligibility was determined through clinical history, physical examination, and routine outpatient evaluation procedures. We recognize that the absence of structured OSA screening represents a methodological limitation. The study was designed as a pragmatic clinical trial reflecting real-world clinical practice in secondary-care public hospital settings, rather than as a controlled laboratory-based sleep study with comprehensive physiological screening.

## 3 Statistical design and analytical strategy

Regarding statistical methodology, our analytical approach focused on within-group pre–post changes and between-group post-intervention comparisons to evaluate short-term treatment-associated improvement and outcome similarity across interventions. While repeated-measures or mixed-effects models offer robust approaches for longitudinal data analysis, they were not employed in this study. The statistical strategy was chosen to provide clinically interpretable comparisons of treatment-related change within a pragmatic clinical trial framework. Importantly, we did not interpret the findings as demonstrating treatment equivalence or superiority, but rather as indicating comparable short-term symptomatic improvement across interventions.

## 4 Statistical reporting and multiple testing

The PSQI global score was predefined as the primary outcome variable. Domain-level subcomponents were analyzed as secondary, exploratory outcomes. Inferential statistical interpretation was therefore centered on the global PSQI score, while subdomain findings were presented to support clinical interpretation rather than to test confirmatory hypotheses. We acknowledge that reporting of F-statistics and formal alpha-level corrections for multiple testing would improve statistical transparency. However, given the exploratory nature of the subdomain analyses and the absence of domain-specific causal or mechanistic claims, these analyses were not positioned as confirmatory statistical endpoints.

## 5 Conclusion

We share the author's emphasis on the importance of rigor in insomnia clinical research. Our study was designed as a pragmatic clinical investigation of integrative therapies within a real-world healthcare context. While this design allows for clinical relevance and generalizability, it also carries methodological limitations, including reliance on subjective outcome measures, absence of objective sleep assessments, and simplified statistical modeling. We believe that our findings contribute to the literature as preliminary clinical evidence supporting the potential role of integrative interventions in chronic insomnia management, while highlighting the need for future trials incorporating objective sleep measures, comprehensive diagnostic screening, longitudinal modeling, and extended follow-up to strengthen the evidence base.

## CRediT authorship contribution statement

**Naruwat Pakdee:** Investigation, Visualization, Writing-original draft, Writing - review and editing. **Nitcha Sribunrieng:** Visualization, Formal analysis. **Ronnachai Poowanna:** Visualization, Formal analysis, writing – original draft, writing – review, and editing.

## Declaration of competing interest

The authors declare that they have no known competing financial interests or personal relationships that could have appeared to influence the work reported in this paper.

